# Titanium vanadium nickel, TiV_0.08_Ni_0.92_

**DOI:** 10.1107/S2414314625001476

**Published:** 2025-02-25

**Authors:** Huizi Liu, Changzeng Fan, Bin Wen, Lifeng Zhang

**Affiliations:** a State Key Laboratory of Metastable Materials Science and Technology Yanshan University,Qinhuangdao 066004 People’s Republic of China; b Hebei Key Lab for Optimizing Metal Product Technology and Performance Yanshan University,Qinhuangdao 066004 People’s Republic of China; c School of Mechanical and Materials Engineering North China University of Technology,Beijing 100144 People’s Republic of China; University of Aberdeen, United Kingdom

**Keywords:** crystal structure, high-pressure sinter­ing, *β* phase, inter­metallic

## Abstract

A single-crystal of the inter­metallic phase TiV_0.08_Ni_0.92_ was obtained by the high-temperature sinter­ing of a mixture of nominal composition Ti_0.9_V_0.1_Ni. The title compound adopts the CsCl structure type with one site solely occupied by Ti and the other by V and Ni with a ratio of 0.08 (7):0.92 (7).

## Graphical abstract

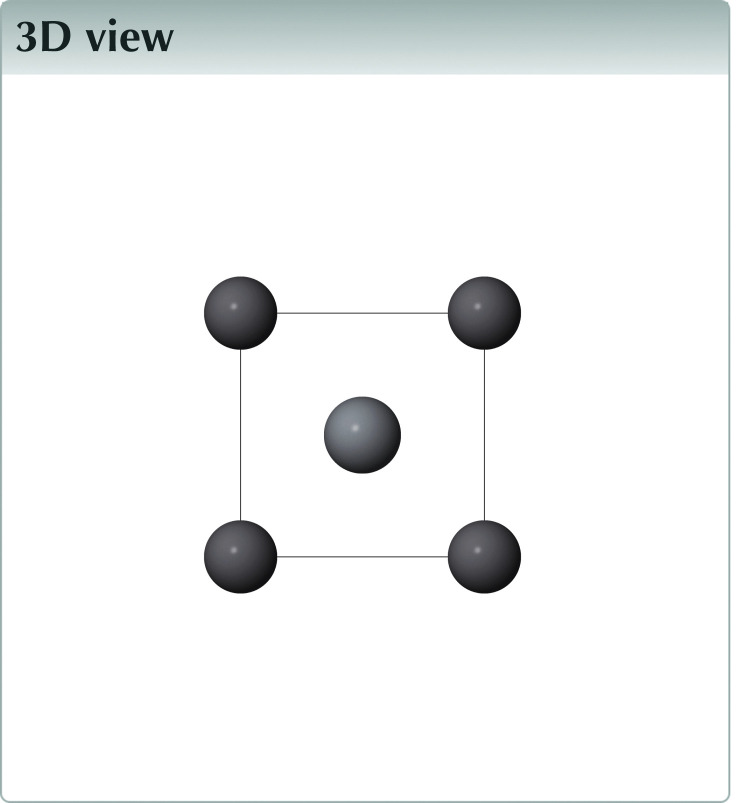

## Structure description

The Ti–V–Ni alloy system has been widely studied for its excellent hydrogen-storage properties. For example, the structure of the Ti_1.4_V_0.6_Ni alloy was studied by powder X-ray diffraction, which identified an icosa­hedral quasicrystal phase (I-phase), fcc-Ti_2_Ni-type phase and bcc-V-based solid-solution phase. The TEM patterns of the I-phase along the fivefold and twofold symmetry axes have been reported (Sun *et al.*, 2015 ▸). Anahara *et al.* (2003 ▸) synthesized the Ti_0.73_V_1.4_Ni_0.27_ alloy, in which Ti was partially replaced by Ni to compare with the parent TiV_1.4_ phase. The PXRD peaks of the Ti_0.73_V_1.4_Ni_0.27_ alloy after heat treatment can be indexed as a b.c.c. solid solution of vanadium and the Ti_2_Ni phase. Iwakura *et al.* (2000 ▸) synthesized TiV_0.9_Ni_0.5_, which is composed of ‘black’ and ‘white’ phases as characterized by X-ray diffraction and electron probe analysis. The black phase is the V-based solid solution, the white phase is a TiNi-based solid solution along with traces of TiNi or Ti_2_Ni-based alloys. The existence of the Ni_3_(Ti_*x*_V_1–*x*_) long-period structure was confirmed by electron diffraction and high-resolution lattice imaging (Zhang *et al.*, 1984 ▸). V_85_Ni_15_ was obtained by dissolving Ni atoms into a vanadium-atom matrix to form a single supersaturated solid solution and V_85_Ni_10_Ti_5_ was obtained by replacing Ni with 5 at% Ti (Jiang *et al.*, 2020 ▸). Souvatzis *et al.* (2010 ▸) prepared the TiNi cubic phase known as the B2 or *β* phase with space group *Pm*
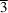
*m.* It can be seen from the literature and databases that previous research on the Ti–V–Ni system only indicated the existence of the bcc structure without any refined structure models.

**Figure 1:**
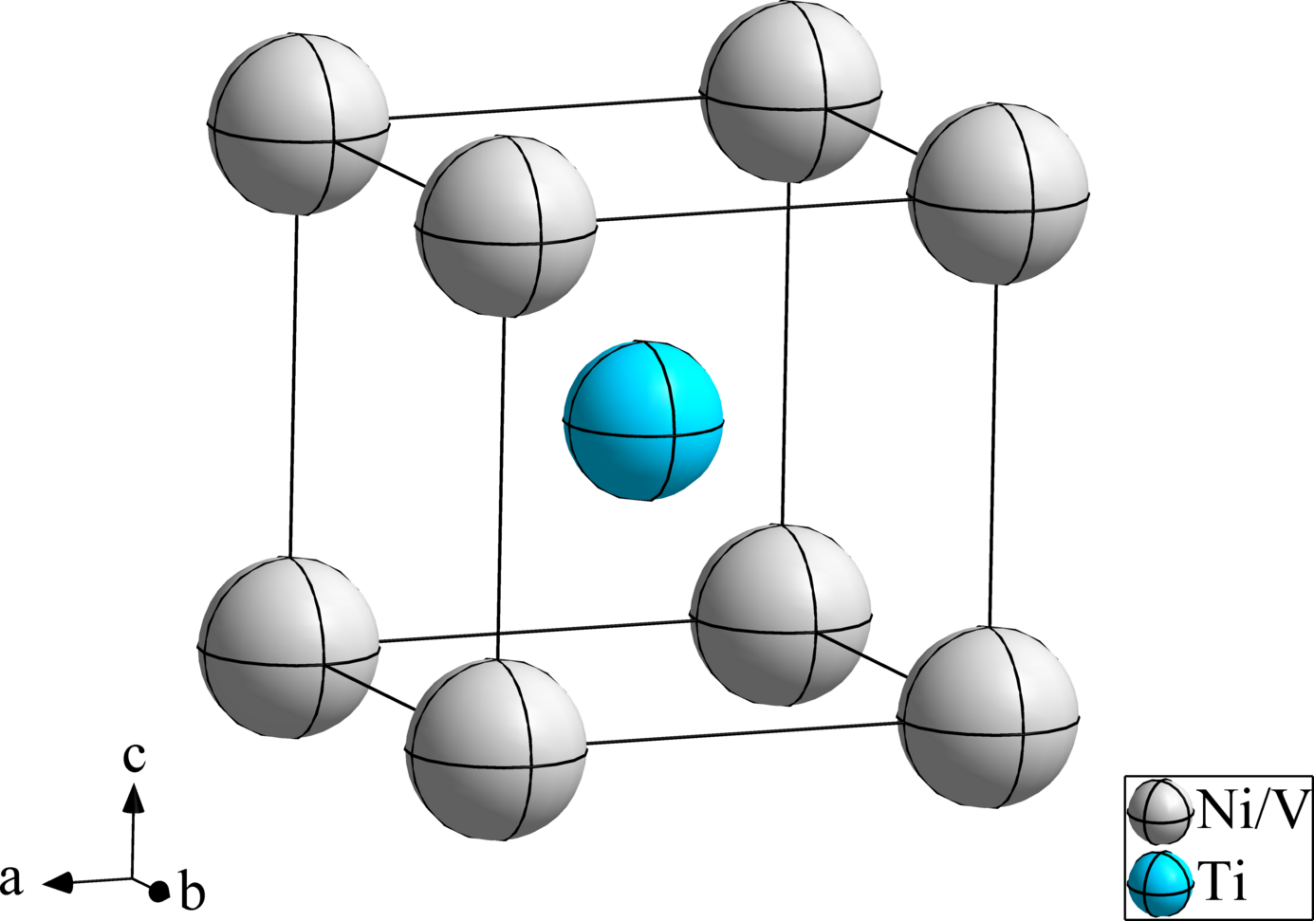
The crystal structure of TiV_0.08_Ni_0.92_, with displacement ellipsoids at the 95% probability level.

**Figure 2:**
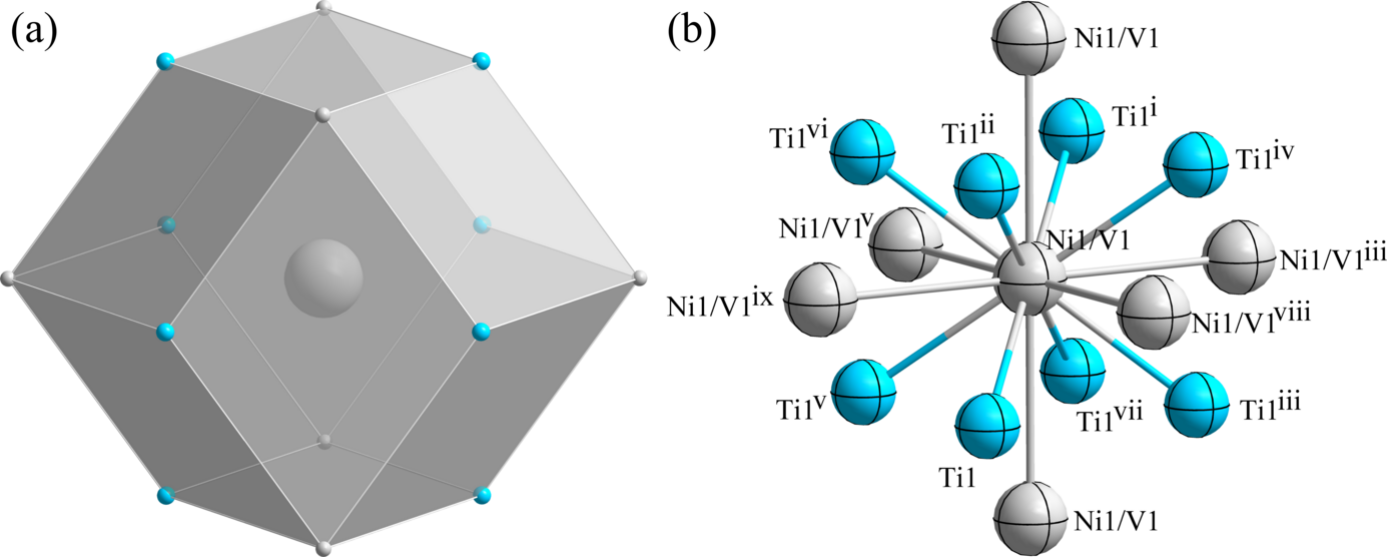
(*a*) The dodeca­hedron formed around the Ni1/V1 atom at the 1*a* Wyckoff site; (*b*) the environment of the Ni1/V1 atom with displacement ellipsoids given at the 95% probability level. [Symmetry codes: (i) *x* − 1, *y* − 1, *z* − 1; (ii) *x* − 1, *y*, *z*; (iii) *x*, *y* − 1, *z*; (iv) *x* − 1, *y* − 1, *z*; (v) *x*, *y*, *z* − 1; (vi) *x* − 1, *y*, *z* − 1; (vii) *x*, *y* − 1, *z* − 1; (viii) *x*, *y*, *z* + 1; (ix) *x*, *y* + 1, *z*.]

**Figure 3:**
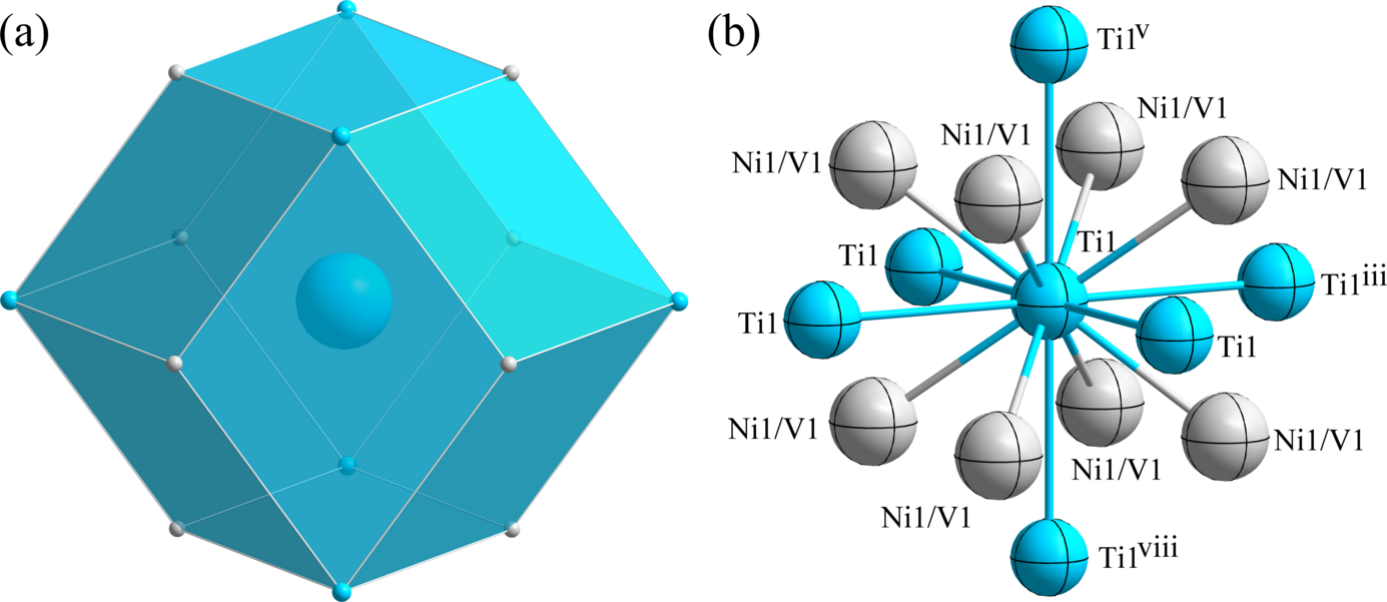
(*a*) The dodeca­hedron formed around the Ti1 atom at the 1*b* site; (*b*) the environment of the Ti1 atom with displacement ellipsoids given at the 95% probability level. [Symmetry codes: (iii) *x*, *y* − 1, *z*; (v) *x*, *y*, *z* − 1; (viii) *x*, *y*, *z* + 1.]

The structure of the title alloy, TiV_0.08_Ni_0.92_, revealed that one site is co-occupied by V and Ni compared with TiNi phase in space-group type *Pm*
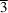
*m.* Fig. 1 ▸ shows the overall atomic distribution in the unit cell of TiV_0.08_Ni_0.92_. Each Ni1/V1 atom is located at a dodeca­hedron (Wyckoff 1*a* site), being surrounded by six Ni1/V1 atoms and eight Ti1 atoms (Fig. 2 ▸). The Ti1 atom (1*b* site) is surrounded by six Ti1 atoms and eight Ni1/V1 atoms, defining the centre of its dodeca­hedron (Fig. 3 ▸). The shortest Ni1/V1 to Ti1 separation is 2.5890 (5) Å and the shortest Ni1/V1 to Ni1/V1 separation is 2.9895 (6) Å.

## Synthesis and crystallization

High-purity titanium powder (indicated purity 99.5%, 0.4043 g), vanadium powder (indicated purity 99.9%, 0.0565 g) and nickel powder (indicated purity 99.9%, 0.5501 g) were mixed in the atomic ratio 0.9:0.1:1 and fully ground in an agate mortar. The mixture was placed into a 5 mm cemented carbide grinding mould and pressed into a tablet at about 6 MPa for 2 min to obtain a cylindrical block without deformations or cracks. The detailed description of the high-pressure sinter­ing experiment using a six-anvil high-temperature and high-pressure apparatus can be found elsewhere (Liu & Fan, 2018 ▸). The sample was pressurized up to 6 GPa and heated to 1623 K for 20 min, cooled to 1173 K and held at that temperature for 1 h. Finally, the furnace power was turned off to rapidly cool to room temperature. Two phases were isolated from two samples from the same batch. According to the complementary EDX results, the chemical composition was refined to be exactly TiV_0.08_Ni_0.92_ originated from sample 1 (see Table S1 of the electronic supporting information, ESI). Another phase of TiV_0.07_Ni_0.93_ with very similar refined composition, was isolated from sample 2, its composition is in accordance with the complementary EDX results also (see Table S2 of the ESI). Different options of refinements for the two phases TiV_*δ*_Ni_1–*δ*_ (*δ* = 0.07, 0.08) are listed in Table S3 of the ESI. The crystal structures of TiV_0.08_Ni_0.92_ and TiV_0.07_Ni_0.93_ are very similar, differing only in atomic proportions at the (Ni/V) site, so the TiV_0.08_Ni_0.92_ phase was selected for the current report. The structure data of TiV_0.07_Ni_0.93_ are summarized in Table S4 of the ESI.

## Refinement

Crystal data, data collection and structure refinement details of TiV_0.08_Ni_0.92_ are summarized in Table 1 ▸. Only one site is co-occupied by Ni and V atoms (Ni1/V1). Site occupation factor (s. o. f.) were refined to 0.08 (7) for V1 and 0.92 (7) for Ni1, assuming full occupancy for each site. Atoms sharing the same site were constrained to have the same coordinates and displacement parameters. The maximum and minimum residual electron densities in the final difference map are located 0.00 Å and 0.78 Å from the atom V1.

## Supplementary Material

Crystal structure: contains datablock(s) I. DOI: 10.1107/S2414314625001476/hb4492sup1.cif

Structure factors: contains datablock(s) I. DOI: 10.1107/S2414314625001476/hb4492Isup2.hkl

Supporting information file. DOI: 10.1107/S2414314625001476/hb4492sup3.docx

CCDC reference: 2424857

Additional supporting information:  crystallographic information; 3D view; checkCIF report

## Figures and Tables

**Table 1:** Experimental details

Crystal data	
Chemical formula	TiV_0.08_Ni_0.92_
*M*_r_	105.96
Crystal system, space group	Cubic, *Pm* 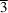 *m*
Temperature (K)	296
*a* (Å)	2.9895 (6)
*V* (Å^3^)	26.72 (2)
*Z*	1
Radiation type	Mo *K*α
μ (mm^−1^)	23.34
Crystal size (mm)	0.10 × 0.08 × 0.06

Data collection	
Diffractometer	Bruker D8 Venture Photon 100 CMOS
Absorption correction	Multi-scan (*SADABS*; Krause *et al.*, 2015 ▸)
*T*_min_, *T*_max_	0.394, 0.746
No. of measured, independent and observed [*I* > 2σ(*I*)] reflections	774, 14, 12
*R*_int_	0.058
(sin θ/λ)_max_ (Å^−1^)	0.626

Refinement	
*R*[*F*^2^ > 2σ(*F*^2^)], *wR*(*F*^2^), *S*	0.022, 0.039, 1.39
No. of reflections	14
No. of parameters	4
Δρ_max_, Δρ_min_ (e Å^−3^)	0.25, −0.20

Computer programs: *APEX3* and *SAINT* (Bruker, 2015 ▸), *SHELXT2014/5* (Sheldrick, 2015*a* ▸), *SHELXL2016/6* (Sheldrick, 2015*b* ▸), *DIAMOND* (Brandenburg & Putz, 2017 ▸) and *publCIF* (Westrip, 2010 ▸).

## full crystallographic data

### Titanium vanadium nickel Crystal data

**Table:**

TiV_0.08_Ni_0.92_	Mo *K*α radiation, λ = 0.71073 Å
*M**_r_* = 105.96	Cell parameters from 433 reflections
Cubic, *P**m*3*m*	θ = 6.8–26.4°
*a* = 2.9895 (6) Å	µ = 23.34 mm^−^^1^
*V* = 26.72 (2) Å^3^	*T* = 296 K
*Z* = 1	Lump, gray
*F*(000) = 50	0.10 × 0.08 × 0.06 mm
*D*_x_ = 6.585 Mg m^−^^3^	

### Titanium vanadium nickel Data collection

**Table:**

Bruker D8 Venture Photon 100 CMOS diffractometer	12 reflections with *I* > 2σ(*I*)
phi and ω scans	*R*_int_ = 0.058
Absorption correction: multi-scan (SADABS; Krause *et al.*, 2015)	θ_max_ = 26.4°, θ_min_ = 6.8°
*T*_min_ = 0.394, *T*_max_ = 0.746	*h* = −3→3
774 measured reflections	*k* = −3→3
14 independent reflections	*l* = −3→3

### Titanium vanadium nickel Refinement

**Table:**

Refinement on *F*^2^	4 parameters
Least-squares matrix: full	0 restraints
*R*[*F*^2^ > 2σ(*F*^2^)] = 0.022	*w* = 1/[σ^2^(*F*_o_^2^) + 0.1191*P*] where *P* = (*F*_o_^2^ + 2*F*_c_^2^)/3
*wR*(*F*^2^) = 0.039	(Δ/σ)_max_ < 0.001
*S* = 1.39	Δρ_max_ = 0.25 e Å^−^^3^
14 reflections	Δρ_min_ = −0.20 e Å^−^^3^

### Titanium vanadium nickel Special details

**Table:**

Geometry. All esds (except the esd in the dihedral angle between two l.s. planes) are estimated using the full covariance matrix. The cell esds are taken into account individually in the estimation of esds in distances, angles and torsion angles; correlations between esds in cell parameters are only used when they are defined by crystal symmetry. An approximate (isotropic) treatment of cell esds is used for estimating esds involving l.s. planes.

### Titanium vanadium nickel Fractional atomic coordinates and isotropic or equivalent isotropic displacement parameters (Å^2^)

**Table:**

	*x*	*y*	*z*	*U*_iso_*/*U*_eq_	Occ. (<1)
Ni1	0.000000	0.000000	0.000000	0.0332 (12)	0.92 (7)
V1	0.000000	0.000000	0.000000	0.0332 (12)	0.08 (7)
Ti1	0.500000	0.500000	0.500000	0.0257 (14)	

### Titanium vanadium nickel Atomic displacement parameters (Å^2^)

**Table:**

	*U*^11^	*U*^22^	*U*^33^	*U*^12^	*U*^13^	*U*^23^
Ni1	0.0332 (12)	0.0332 (12)	0.0332 (12)	0.000	0.000	0.000
V1	0.0332 (12)	0.0332 (12)	0.0332 (12)	0.000	0.000	0.000
Ti1	0.0257 (14)	0.0257 (14)	0.0257 (14)	0.000	0.000	0.000

### Titanium vanadium nickel Geometric parameters (Å, º)

**Table:**

Ni1—Ti1^i^	2.5890 (5)	V1—Ti1^i^	2.5890 (5)
Ni1—Ti1	2.5890 (5)	V1—Ti1	2.5890 (5)
Ni1—Ti1^ii^	2.5890 (5)	V1—Ti1^ii^	2.5890 (5)
Ni1—Ti1^iii^	2.5890 (5)	V1—Ti1^vii^	2.5890 (5)
Ni1—Ti1^iv^	2.5890 (5)	V1—Ti1^vi^	2.5890 (5)
Ni1—Ti1^v^	2.5890 (5)	V1—Ti1^v^	2.5890 (5)
Ni1—Ti1^vi^	2.5890 (5)	V1—Ti1^iv^	2.5890 (5)
Ni1—Ti1^vii^	2.5890 (5)	V1—Ti1^iii^	2.5890 (5)
Ni1—Ni1^iii^	2.9895 (6)	Ti1—Ti1^viii^	2.9895 (6)
Ni1—Ni1^v^	2.9895 (6)	Ti1—Ti1^v^	2.9895 (6)
Ni1—Ni1^viii^	2.9895 (6)	Ti1—Ti1^iii^	2.9895 (6)
Ni1—Ni1^ix^	2.9895 (6)		
			
Ti1^i^—Ni1—Ti1	180.0	Ti1^vii^—V1—Ti1^vi^	109.5
Ti1^i^—Ni1—Ti1^ii^	109.5	Ti1^i^—V1—Ti1^v^	109.5
Ti1—Ni1—Ti1^ii^	70.5	Ti1—V1—Ti1^v^	70.5
Ti1^i^—Ni1—Ti1^iii^	109.5	Ti1^ii^—V1—Ti1^v^	109.5
Ti1—Ni1—Ti1^iii^	70.5	Ti1^vii^—V1—Ti1^v^	70.5
Ti1^ii^—Ni1—Ti1^iii^	109.5	Ti1^vi^—V1—Ti1^v^	70.5
Ti1^i^—Ni1—Ti1^iv^	70.5	Ti1^i^—V1—Ti1^iv^	70.5
Ti1—Ni1—Ti1^iv^	109.5	Ti1—V1—Ti1^iv^	109.5
Ti1^ii^—Ni1—Ti1^iv^	70.5	Ti1^ii^—V1—Ti1^iv^	70.5
Ti1^iii^—Ni1—Ti1^iv^	70.5	Ti1^vii^—V1—Ti1^iv^	109.5
Ti1^i^—Ni1—Ti1^v^	109.5	Ti1^vi^—V1—Ti1^iv^	109.5
Ti1—Ni1—Ti1^v^	70.5	Ti1^v^—V1—Ti1^iv^	180.0
Ti1^ii^—Ni1—Ti1^v^	109.5	Ti1^i^—V1—Ti1^iii^	109.5
Ti1^iii^—Ni1—Ti1^v^	109.5	Ti1—V1—Ti1^iii^	70.5
Ti1^iv^—Ni1—Ti1^v^	180.0	Ti1^ii^—V1—Ti1^iii^	109.5
Ti1^i^—Ni1—Ti1^vi^	70.5	Ti1^vii^—V1—Ti1^iii^	70.5
Ti1—Ni1—Ti1^vi^	109.5	Ti1^vi^—V1—Ti1^iii^	180.0
Ti1^ii^—Ni1—Ti1^vi^	70.5	Ti1^v^—V1—Ti1^iii^	109.5
Ti1^iii^—Ni1—Ti1^vi^	180.0	Ti1^iv^—V1—Ti1^iii^	70.5
Ti1^iv^—Ni1—Ti1^vi^	109.5	Ni1^x^—Ti1—Ni1	180.0
Ti1^v^—Ni1—Ti1^vi^	70.5	Ni1^x^—Ti1—Ni1^xi^	109.5
Ti1^i^—Ni1—Ti1^vii^	70.5	Ni1—Ti1—Ni1^xi^	70.5
Ti1—Ni1—Ti1^vii^	109.5	Ni1^x^—Ti1—Ni1^ix^	109.5
Ti1^ii^—Ni1—Ti1^vii^	180.0	Ni1—Ti1—Ni1^ix^	70.5
Ti1^iii^—Ni1—Ti1^vii^	70.5	Ni1^xi^—Ti1—Ni1^ix^	109.5
Ti1^iv^—Ni1—Ti1^vii^	109.5	Ni1^x^—Ti1—Ni1^xii^	70.5
Ti1^v^—Ni1—Ti1^vii^	70.5	Ni1—Ti1—Ni1^xii^	109.5
Ti1^vi^—Ni1—Ti1^vii^	109.5	Ni1^xi^—Ti1—Ni1^xii^	70.5
Ti1^i^—Ni1—Ni1^iii^	54.7	Ni1^ix^—Ti1—Ni1^xii^	70.5
Ti1—Ni1—Ni1^iii^	125.3	Ni1^x^—Ti1—Ni1^viii^	109.5
Ti1^ii^—Ni1—Ni1^iii^	125.3	Ni1—Ti1—Ni1^viii^	70.529 (1)
Ti1^iii^—Ni1—Ni1^iii^	54.7	Ni1^xi^—Ti1—Ni1^viii^	109.5
Ti1^iv^—Ni1—Ni1^iii^	54.7	Ni1^ix^—Ti1—Ni1^viii^	109.5
Ti1^v^—Ni1—Ni1^iii^	125.3	Ni1^xii^—Ti1—Ni1^viii^	180.0
Ti1^vi^—Ni1—Ni1^iii^	125.3	Ni1^x^—Ti1—Ni1^xiii^	70.5
Ti1^vii^—Ni1—Ni1^iii^	54.7	Ni1—Ti1—Ni1^xiii^	109.5
Ti1^i^—Ni1—Ni1^v^	54.7	Ni1^xi^—Ti1—Ni1^xiii^	70.5
Ti1—Ni1—Ni1^v^	125.3	Ni1^ix^—Ti1—Ni1^xiii^	180.0
Ti1^ii^—Ni1—Ni1^v^	125.3	Ni1^xii^—Ti1—Ni1^xiii^	109.5
Ti1^iii^—Ni1—Ni1^v^	125.3	Ni1^viii^—Ti1—Ni1^xiii^	70.5
Ti1^iv^—Ni1—Ni1^v^	125.3	Ni1^x^—Ti1—Ni1^xiv^	70.5
Ti1^v^—Ni1—Ni1^v^	54.7	Ni1—Ti1—Ni1^xiv^	109.5
Ti1^vi^—Ni1—Ni1^v^	54.7	Ni1^xi^—Ti1—Ni1^xiv^	180.0
Ti1^vii^—Ni1—Ni1^v^	54.7	Ni1^ix^—Ti1—Ni1^xiv^	70.5
Ni1^iii^—Ni1—Ni1^v^	90.0	Ni1^xii^—Ti1—Ni1^xiv^	109.5
Ti1^i^—Ni1—Ni1^viii^	125.3	Ni1^viii^—Ti1—Ni1^xiv^	70.5
Ti1—Ni1—Ni1^viii^	54.7	Ni1^xiii^—Ti1—Ni1^xiv^	109.5
Ti1^ii^—Ni1—Ni1^viii^	54.7	Ni1^x^—Ti1—Ti1^viii^	54.7
Ti1^iii^—Ni1—Ni1^viii^	54.7	Ni1—Ti1—Ti1^viii^	125.3
Ti1^iv^—Ni1—Ni1^viii^	54.7	V1—Ti1—Ti1^viii^	125.3
Ti1^v^—Ni1—Ni1^viii^	125.3	Ni1^xi^—Ti1—Ti1^viii^	125.3
Ti1^vi^—Ni1—Ni1^viii^	125.3	Ni1^ix^—Ti1—Ti1^viii^	125.3
Ti1^vii^—Ni1—Ni1^viii^	125.3	Ni1^xii^—Ti1—Ti1^viii^	125.3
Ni1^iii^—Ni1—Ni1^viii^	90.0	Ni1^viii^—Ti1—Ti1^viii^	54.7
Ni1^v^—Ni1—Ni1^viii^	180.0	Ni1^xiii^—Ti1—Ti1^viii^	54.7
Ti1^i^—Ni1—Ni1^ix^	125.3	Ni1^xiv^—Ti1—Ti1^viii^	54.7
Ti1—Ni1—Ni1^ix^	54.7	Ni1^x^—Ti1—Ti1^v^	125.3
Ti1^ii^—Ni1—Ni1^ix^	54.7	Ni1—Ti1—Ti1^v^	54.7
Ti1^iii^—Ni1—Ni1^ix^	125.3	Ni1^xi^—Ti1—Ti1^v^	54.7
Ti1^iv^—Ni1—Ni1^ix^	125.3	Ni1^ix^—Ti1—Ti1^v^	54.7
Ti1^v^—Ni1—Ni1^ix^	54.7	Ni1^xii^—Ti1—Ti1^v^	54.7
Ti1^vi^—Ni1—Ni1^ix^	54.7	Ni1^viii^—Ti1—Ti1^v^	125.3
Ti1^vii^—Ni1—Ni1^ix^	125.3	Ni1^xiii^—Ti1—Ti1^v^	125.3
Ni1^iii^—Ni1—Ni1^ix^	180.0	Ni1^xiv^—Ti1—Ti1^v^	125.3
Ni1^v^—Ni1—Ni1^ix^	90.0	Ti1^viii^—Ti1—Ti1^v^	180.0
Ni1^viii^—Ni1—Ni1^ix^	90.0	Ni1^x^—Ti1—Ti1^iii^	125.3
Ti1^i^—V1—Ti1	180.0	Ni1—Ti1—Ti1^iii^	54.7
Ti1^i^—V1—Ti1^ii^	109.5	Ni1^xi^—Ti1—Ti1^iii^	54.7
Ti1—V1—Ti1^ii^	70.5	Ni1^ix^—Ti1—Ti1^iii^	125.3
Ti1^i^—V1—Ti1^vii^	70.5	Ni1^xii^—Ti1—Ti1^iii^	125.3
Ti1—V1—Ti1^vii^	109.5	Ni1^viii^—Ti1—Ti1^iii^	54.7
Ti1^ii^—V1—Ti1^vii^	180.0	Ni1^xiii^—Ti1—Ti1^iii^	54.7
Ti1^i^—V1—Ti1^vi^	70.5	Ni1^xiv^—Ti1—Ti1^iii^	125.3
Ti1—V1—Ti1^vi^	109.5	Ti1^viii^—Ti1—Ti1^iii^	90.0
Ti1^ii^—V1—Ti1^vi^	70.5	Ti1^v^—Ti1—Ti1^iii^	90.0

Symmetry codes: (i) *x*−1, *y*−1, *z*−1; (ii) *x*−1, *y*, *z*; (iii) *x*, *y*−1, *z*; (iv) *x*−1, *y*−1, *z*; (v) *x*, *y*, *z*−1; (vi) *x*−1, *y*, *z*−1; (vii) *x*, *y*−1, *z*−1; (viii) *x*, *y*, *z*+1; (ix) *x*, *y*+1, *z*; (x) *x*+1, *y*+1, *z*+1; (xi) *x*+1, *y*, *z*; (xii) *x*+1, *y*+1, *z*; (xiii) *x*+1, *y*, *z*+1; (xiv) *x*, *y*+1, *z*+1.
