# Supplementary material for: Titanium vanadium nickel, TiV0.08Ni0.92
Source: IUCrdata. 2025 Feb 25;10(Pt 2):x250147. doi: 10.1107/S2414314625001476 (PMC11904629; doi:10.1107/S2414314625001476)
Supplement: Supplementary file 3 [file x-10-x250147-sup3.docx]

**SUPPLEMENTARY MATERIALS:**

**Crystal structure of TiV_0.08_Ni_0.92_**

**Huizi Liu**^a^**,** **Changzeng Fan**^a,b,^***, Bin Wen**^a^**,** and **Lifeng Zhang** ^a,c^

^a^ State Key Laboratory of Metastable Materials Science and Technology, Yanshan University,

Qinhuangdao 066004, People’s Republic of China

^b^ Hebei Key Lab for Optimizing Metal Product Technology and Performance, Yanshan University, Qinhuangdao, Hebei 066004, People’s Republic of China

^c^ School of Mechanical and Materials Engineering, North China University of Technology, Beijing 100144, People's Republic of China

*Correspondence email: [chzfan@ysu.edu.cn](mailto:chzfan@ysu.edu.cn)

The chemical compositions were examined quantitatively by energy dispersive X-ray spectroscopy (EDX) analysis attached to a Hitachi S-3400N SEM for the purpose of guiding the crystal structure refinement. The examined points and areas of sample 1 (sample 2) are designated in Fig. S1 (Fig. S2), and the corresponding results are listed in Table S1 (Table S2). The deviation relative to the results of refinement of chemical composition is probably caused by the tilt of the single crystal surface to the incident beam. In addition, the conductive adhesives and glues may also result in the detected impurity elements of carbon and oxygen. For ease of reading, the atomic ratio of Ti, V and Ni was calculated and shown in the last column of Table S1 (Table S2).


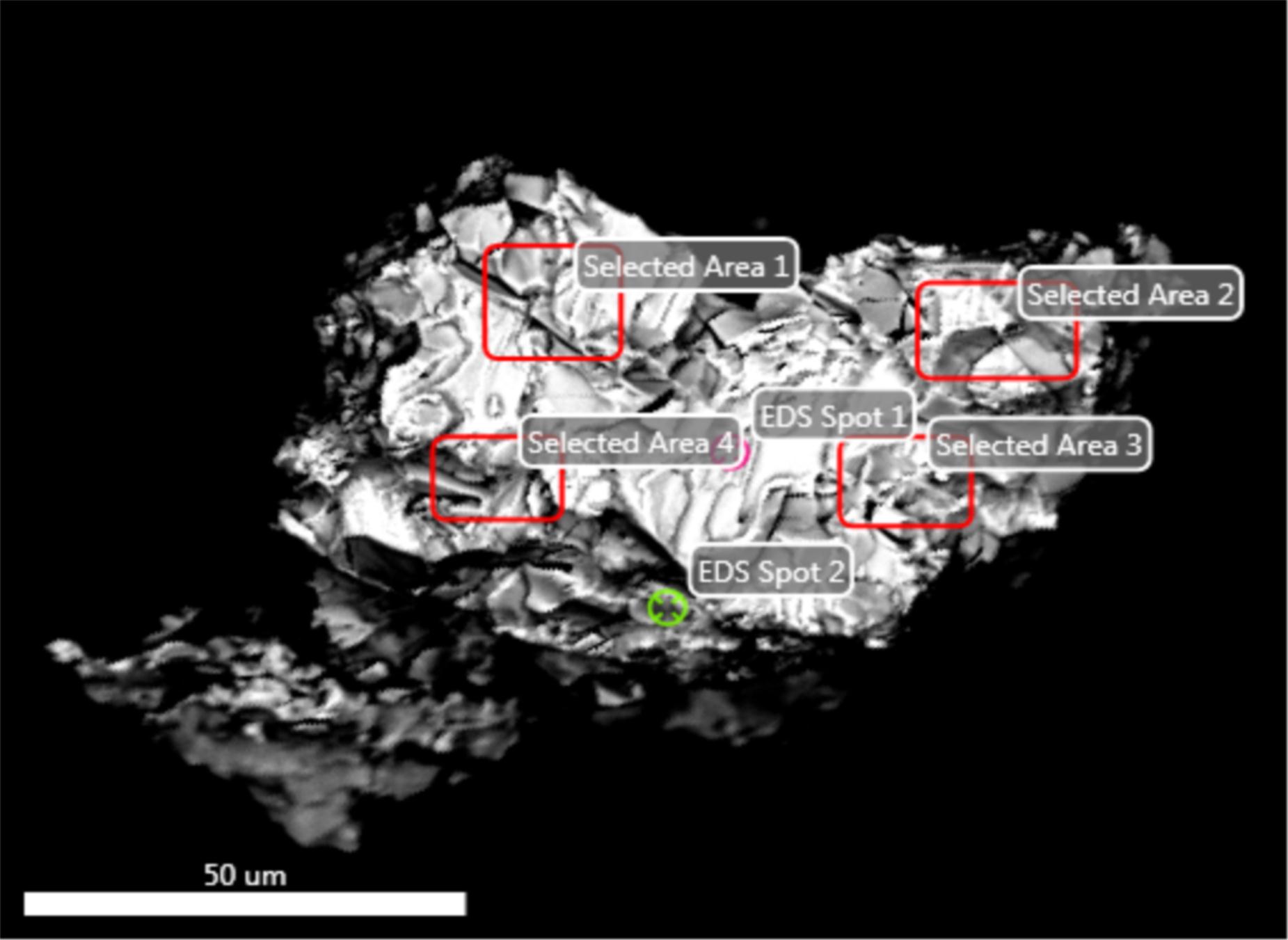


Fig. S1 Sample 1 with selected spots and areas for EDX analysis

**Table S1 EDX results for selected points and areas as designated in Fig. S1**

|  | Element | Weight (%) | Atomic (%) | Error (%) | Ti : V : Ni |
| --- | --- | --- | --- | --- | --- |
| Spot1 | C K | 8.92 | 29.45 | 10.20 | 1:0.13:1.26 |
|  | O K | 1.92 | 4.76 | 18.18 |  |
|  | TiK | 33.16 | 27.47 | 2.00 |  |
|  | V K | 4.61 | 3.59 | 4.52 |  |
|  | NiK | 51.39 | 34.73 | 2.32 |  |
| Spot2 | C K | 8.42 | 28.17 | 11.63 | 1:0.23:0.92 |
|  | O K | 1.25 | 3.13 | 18.90 |  |
|  | TiK | 37.96 | 31.85 | 2.17 |  |
|  | V K | 9.47 | 7.47 | 3.58 |  |
|  | NiK | 42.91 | 29.38 | 2.92 |  |
| Area1 | C K | 9.16 | 29.75 | 9.95 | 1:0.17:0.92 |
|  | O K | 1.92 | 4.69 | 14.58 |  |
|  | TiK | 38.47 | 31.33 | 1.86 |  |
|  | V K | 7.10 | 5.44 | 2.89 |  |
|  | NiK | 43.34 | 28.79 | 2.41 |  |
| Area2 | C K | 16.80 | 43.96 | 9.14 | 1:0.19:0.96 |
|  | O K | 4.89 | 9.60 | 13.11 |  |
|  | TiK | 32.85 | 21.55 | 1.83 |  |
|  | V K | 6.74 | 4.16 | 2.83 |  |
|  | NiK | 38.73 | 20.73 | 2.36 |  |
| Area3 | C K | 12.52 | 37.39 | 9.79 | 1:0.12:1.23 |
|  | O K | 2.66 | 5.96 | 17.65 |  |
|  | TiK | 32.21 | 24.12 | 1.98 |  |
|  | V K | 4.06 | 2.86 | 5.16 |  |
|  | NiK | 48.55 | 29.66 | 2.33 |  |
| Area4 | C K | 11.69 | 35.60 | 9.70 | 1:0.16:1.07 |
|  | O K | 2.37 | 5.43 | 15.27 |  |
|  | TiK | 34.58 | 26.40 | 1.91 |  |
|  | V K | 6.01 | 4.32 | 3.12 |  |
|  | NiK | 45.35 | 28.25 | 2.36 |  |


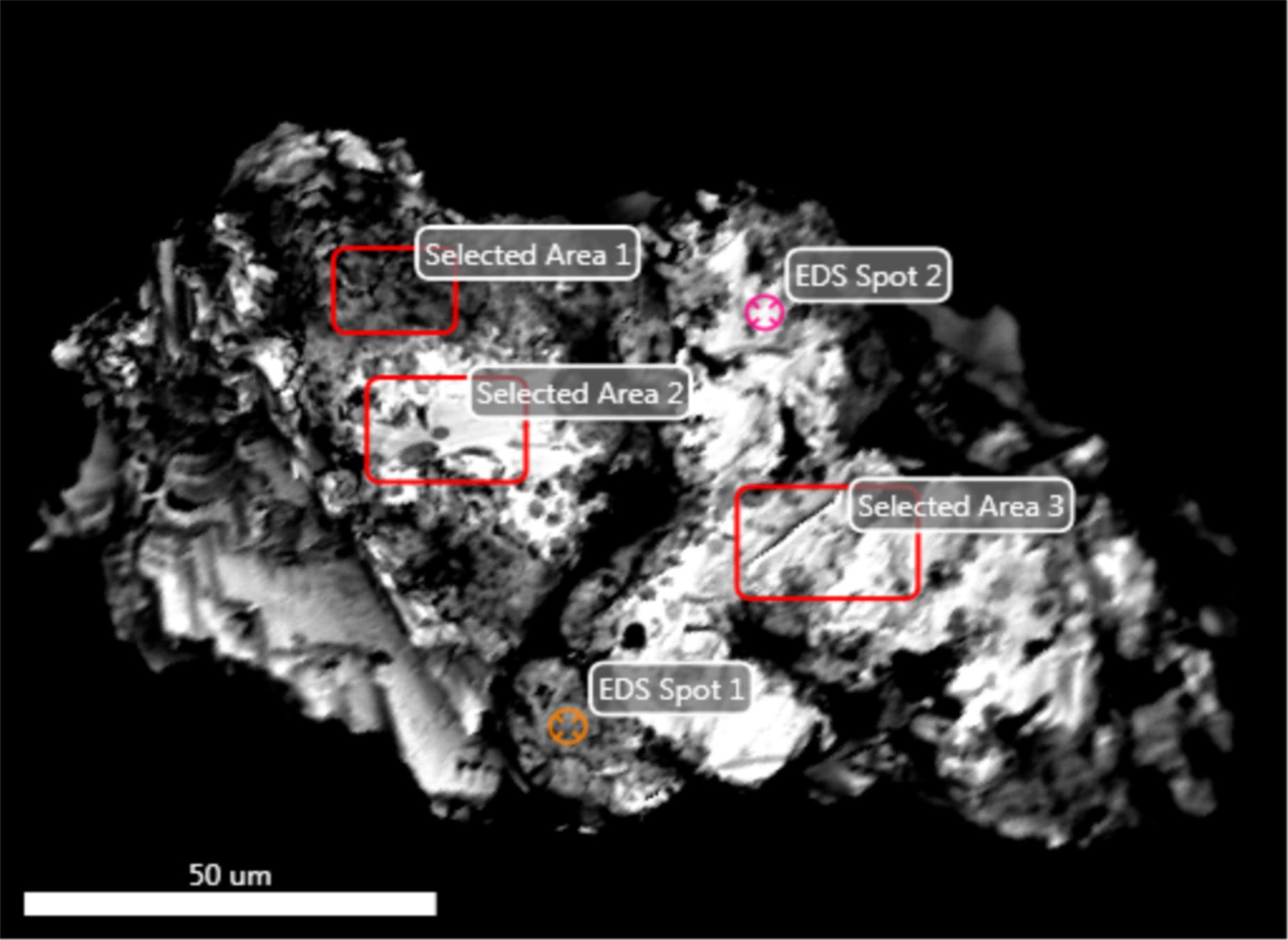


Fig. S2 Sample 2 with selected spots and areas for EDX analysis

**Table S2 EDX results for selected points and areas as designated in Fig. S2**

|  | Element | Weight (%) | Atomic (%) | Error (%) | Ti : V : Ni |
| --- | --- | --- | --- | --- | --- |
| Spot1 | C K | 6.83 | 17.76 | 8.41 | 1:0.01:0.05 |
|  | N K | 12.26 | 27.35 | 8.17 |  |
|  | O K | 2.01 | 3.92 | 23.11 |  |
|  | TiK | 73.78 | 48.15 | 1.46 |  |
|  | V K | 0.93 | 0.57 | 10.68 |  |
|  | NiK | 4.20 | 2.24 | 5.22 |  |
| Spot2 | C K | 8.69 | 29.65 | 10.10 | 1:0.17:1.03 |
|  | TiK | 37.45 | 32.04 | 1.91 |  |
|  | V K | 6.77 | 5.45 | 2.96 |  |
|  | NiK | 47.08 | 32.86 | 2.39 |  |
| Area1 | C K | 15.17 | 29.32 | 7.37 | 1:0.05:0.08 |
|  | N K | 18.84 | 31.22 | 8.88 |  |
|  | O K | 8.26 | 11.98 | 13.73 |  |
|  | TiK | 50.52 | 24.48 | 1.56 |  |
|  | V K | 2.55 | 1.16 | 3.73 |  |
|  | NiK | 4.66 | 1.84 | 4.05 |  |
| Area2 | C K | 8.81 | 26.64 | 9.77 | 1:0.17:0.89 |
|  | N K | 3.52 | 9.12 | 10.84 |  |
|  | O K | 2.53 | 5.74 | 19.26 |  |
|  | TiK | 37.41 | 28.35 | 1.85 |  |
|  | V K | 6.85 | 4.88 | 2.95 |  |
|  | NiK | 40.87 | 25.27 | 2.41 |  |
| Area3 | C K | 12.46 | 35.89 | 9.91 | 1:0.20:0.95 |
|  | O K | 2.49 | 5.38 | 14.13 |  |
|  | SiK | 5.47 | 6.74 | 6.40 |  |
|  | TiK | 33.36 | 24.10 | 1.90 |  |
|  | V K | 7.22 | 4.90 | 2.89 |  |
|  | NiK | 39.01 | 22.99 | 2.38 |  |

**Table S3** Different options of refinement and the resulting refined chemical compositions

|  | location | | compositions | | | R1 |
| --- | --- | --- | --- | --- | --- | --- |
| TiV_0.08_Ni_0.92_ | 1*a* | 1*b* | Ti | V | Ni |  |
|  | Ti | Ni | 0.99984 |  | 0.99984 | 2.48 |
|  | Ti/V | Ni | 0.720535 | 0.279305 | 0.99984 | 2.27 |
|  | **Ti** | **Ni/V** | **0.99984** | **0.0819269** | **0.917913** | **2.23** |
|  | Ti/V | Ni/V | Refinement unstable | | |  |
|  | Ti/V | Ni/Ti | Refinement unstable | | |  |
|  | Ti/Ni | Ni/V | Refinement unstable | | |  |
| TiV_0.07_Ni_0.93_ | Ti | Ni | 0.99984 |  | 0.99984 | 3.77 |
|  | Ti/V | Ni | 0.777366 | 0.222474 | 0.99984 | 3.63 |
|  | **Ti** | **Ni/V** | **0.99984** | **0.0694689** | **0.930371** | **3.59** |
|  | Ti/V | Ni/V | Refinement unstable | | |  |
|  | Ti/V | Ni/Ti | Refinement unstable | | |  |
|  | Ti/Ni | Ni/V | Refinement unstable | | |  |

**Table S4** Experimental details of TiV_0.07_Ni_0.93_ phase

|  | TiV_0.07_Ni_0.93_ |
| --- | --- |
| Crystal data | |
| Chemical formula | TiV_0.07_Ni_0.93_ |
| *M*_r_ | 106.05 |
| Crystal system, space group | Cubic, *Pm*$\bar{\text{3}}$*m* |
| Temperature (K) | 296 |
| *a* (Å) | 2.995 (3) |
| *V* (Å^3^) | 26.87 (8) |
| *Z* | 1 |
| Radiation type | Mo-K*α* |
| µ (mm^-1^) | 23.32 |
| Crystal size (mm) | 0.12×0.10×0.10 |
| Data collection | |
| Diffractometer | Bruker D8 Venture Photon 100 COMS |
| Absorption correction | multi-scan  (SADABS; Krause *et al*., 2015) |
| *T*_min_, *T*_max_ | 0.363, 0.746 |
| No. of measured, independent and  observed [*I* > 2σ(*I*)] reflections | 609, 14, 11 |
| *R_int_* | 0.088 |
| (sin θ/λ)_max_ (Å^−1^) | 0.625 |
| Refinement | |
| *R*[*F*^2^ > 2σ(*F*^2^)], *wR*(*F*^2^), *S* | 0.036, 0.084, 1.18 |
| No. of reflections | 14 |
| No. of parameters | 4 |
| Δ*ρ*_max_, Δ*ρ*_min_ (e Å^−3^) | 0.48, -0.44 |

Computer programs: *APEX3* (Bruker, 2015), *APEX3* and *SAINT* (Bruker, 2015), *SHELXT* 2014/5 (Sheldrick, 2015), *SHELXL2016*/6 (Sheldrick, 2015), *publCIF* (Westrip, 2010).
